# Clinical Static Balance Assessment: A Narrative Review of Traditional and IMU-Based Posturography in Older Adults and Individuals with Incomplete Spinal Cord Injury

**DOI:** 10.3390/s23218881

**Published:** 2023-11-01

**Authors:** Alireza Noamani, Negar Riahi, Albert H. Vette, Hossein Rouhani

**Affiliations:** 1 Department of Mechanical Engineering, University of Alberta, Edmonton, AB T6G 1H9, Canada; noamani@ualberta.ca (A.N.); riahi@ualberta.ca (N.R.); vette@ualberta.ca (A.H.V.); 2 Department of Biomedical Engineering, University of Alberta, Edmonton, AB T6G 1H9, Canada; 3 Glenrose Rehabilitation Hospital, Alberta Health Services, Edmonton, AB T5G 0B7, Canada

**Keywords:** balance, older adults, incomplete spinal cord injury, IMU-based posturography, postural stability, posturography, static balance assessment

## Abstract

Maintaining a stable upright posture is essential for performing activities of daily living, and impaired standing balance may impact an individual’s quality of life. Therefore, accurate and sensitive methods for assessing static balance are crucial for identifying balance impairments, understanding the underlying mechanisms of the balance deficiencies, and developing targeted interventions to improve standing balance and prevent falls. This review paper first explores the methods to quantify standing balance. Then, it reviews traditional posturography and recent advancements in using wearable inertial measurement units (IMUs) to assess static balance in two populations: older adults and those with incomplete spinal cord injury (iSCI). The inclusion of these two groups is supported by their large representation among individuals with balance impairments. Also, each group exhibits distinct aspects in balance assessment due to diverse underlying causes associated with aging and neurological impairment. Given the high vulnerability of both demographics to balance impairments and falls, the significance of targeted interventions to improve standing balance and mitigate fall risk becomes apparent. Overall, this review highlights the importance of static balance assessment and the potential of emerging methods and technologies to improve our understanding of postural control in different populations.

## 1. Introduction

Falls are among the most frequent causes of injury in older adults and individuals with neuromuscular impairments [1,2]. Previous studies have shown that impairment of gait or balance is the most consistent predictor of falls [3]. Hence, those with balance impairment are at a high risk of falling, and thus, the ability to maintain balance during daily life has been used to evaluate fall risk in these individuals and the outcome of rehabilitative interventions [4,5]. Up to one-third of seniors fall at least once a year [6], with over 50% of fallers reporting multiple falls [7]. The fall rate among older adults grows with age [8], making falls the fifth leading cause of death in older adults [9]. At the same time, the literature has reported a high occurrence of falling incidences among individuals with neurological conditions, such as spinal cord injury (SCI). Up to 78% of ambulatory individuals with SCI experience at least one fall post-rehabilitation [10,11,12]. Falls can lead to injuries and hospitalization [10], restriction in community participation [11,13,14], and the development of a fear of falling [15]. In addition, up to 30% of individuals with a recent SCI and most individuals with an incomplete SCI (iSCI) are able to regain partial balance and walking ability after the first year post-injury [16]. However, a significant challenge for individuals with iSCI is to maintain postural stability while recovering walking function [17]. iSCI affects the ability to stand stably and perform daily activities [18]. Maintaining standing balance and restoring the ability to walk are the top priorities for individuals with iSCI [16,19,20]. One of the major factors contributing to falls in this population is the loss of balance [14,18], highlighting the lack of effective postural control in individuals with iSCI. The development of fall prevention strategies is associated with effective postural control. Hence, the implementation of outcome measures that identify the balance abilities of individuals with neuromuscular impairments, such as iSCI, can lead to more effective rehabilitation [16]. In this context, understanding the underlying mechanisms of how postural control is regulated post-impairment is of significant importance.

Physical therapists oftentimes use standard observational rating scales to evaluate balance. The concurrent validity of observational balance scales for older adults and individuals with neurological conditions, e.g., the Berg Balance Scale (BBS) or Mini Balance Evaluation System Test (Mini-BEST), has been studied in the past. However, they tend to be subjective and may provide limited information for understanding the potential underlying mechanisms for the balance deficiencies [16,21,22], highlighting a need for a quantitative approach to evaluate standing balance. Hence, an instrumented version of such tests can provide objective balance evaluation by measuring how balance is impaired, with high sensitivity in the identification of mild balance alterations [23]. Therapists may use such objective measures to track subtle changes in postural control over time and precisely focus the therapy on underlying causes [23].

The objective assessment of balance has been investigated based on the reaction forces from the ground and the body segments’ movement (e.g., joint angles) during standing, measured via force plates and motion capture systems, respectively [24,25]. Nevertheless, the implementation of in-lab equipment for clinical research and practice has not become practical due to the requirement of expensive equipment and dedicated lab space. Wearable inertial measurement units (IMUs) have been used as an alternative for obtaining sensitive measures of balance in populations with neurological conditions, such as individuals with Parkinson’s disease [26], traumatic brain injury [23], and SCI [27]. The expanding use of traditional and IMU-based posturography for clinical static balance assessment in individuals with neurological conditions highlights a pressing need for a comprehensive review that synthesizes the existing literature, provides insights into the state-of-the-art practices in this field, and identifies critical knowledge gaps. This review paper then specifically focuses on the balance assessment of older adults and individuals with iSCI. The global older adult population, around 9% of the world’s population in 2019, is expected to double by 2050 [28]. Also, there are approximately 285,000 individuals with SCI in the United States, with 66.7% experiencing incomplete SCI [29]. In both cases, balance assessment plays a vital role. For older adults, it helps detect balance issues early, allowing for targeted interventions to prevent falls and promote independence and well-being. Similarly, in individuals with iSCI, a comprehensive balance assessment provides valuable insights into specific deficits, guiding personalized interventions to reduce fall risk and improve functional outcomes. Also, older adults and those with iSCI respond to different aspects of balance assessment due to the distinct underlying causes and factors affecting balance in each group. On the one hand, in older adults, balance assessment takes into account age-related factors, such as declines in physical strength, flexibility, and sensorimotor integration. On the other hand, in individuals with iSCI, balance assessment focuses on understanding the impact of the spinal cord injury on motor control, muscle weakness, sensory deficits, and altered proprioception. Tailoring the assessment to the specific needs and challenges of each group enables professionals to develop targeted interventions and strategies to improve balance and reduce fall risk effectively.

**Figure 1:**
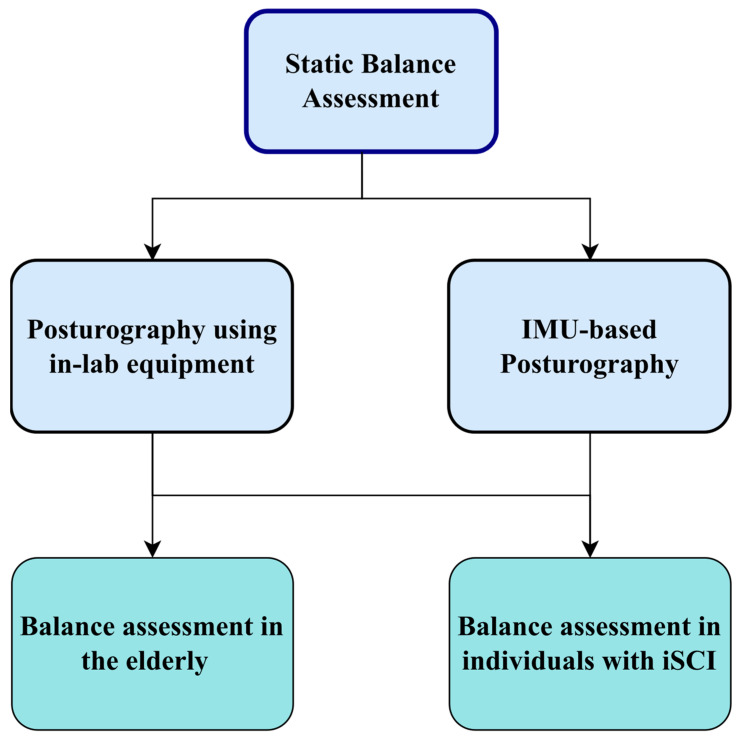
Structure of the review article with an overview of key sections.

In summary, these two groups represent populations with unique challenges related to balance, making them particularly relevant for investigating the effectiveness and applicability of posturography techniques. By focusing on these specific populations, this review aims to provide targeted insights and recommendations for optimizing balance assessment in these high-risk groups. In particular, we discuss methods of quantifying standing balance in Section 2. Then, standard posturographic measures introduced by the literature based on the measurements of in-lab equipment and wearable IMUs are discussed in Section 3.1 and Section 3.2, respectively. As justified above, this review will focus on two groups with potentially impaired standing balance: older adults and individuals with iSCI. In Section 4 and Section 5, we discuss how the literature has benefited from traditional posturography using motion capture and force platform, as well as recent advancements in wearable IMUs to characterize postural control during quiet standing for older adults and individuals with iSCI, respectively. The overview of the key sections of this paper can be seen in Figure 1.

## 2. Quantification of Standing Balance

Bipedal human standing, from a mechanical point of view, is inherently unstable [30] since its stability requires the body, a large object consisting of many segments, to remain in an erect posture, with the body’s center of mass (COM) located within a relatively small base of support (BOS) [31]. Successful stabilization of body posture requires coordination among different components of the body’s neuro–musculo–skeletal system, including sensory modalities, joints, and muscles [32]. Studying the mechanisms responsible for postural control is of significant interest to many researchers. Spontaneous sway during quiet standing has been widely used to investigate human postural control by analyzing the body’s center of pressure (COP) trajectory [33,34], the body’s COM trajectory [35,36], or the ankle and hip joint motions [30,37].

Standing balance represents an individual’s ability to maintain the body’s COM and COP within the boundaries of an established BOS [38,39,40]. The COM represents the centroid of all segment masses of the body, whereas the COP represents the point of application of the ground reaction forces (GRFs) [39]. Researchers have extensively used both COM and COP to evaluate standing balance performance, identify impaired balance due to underlying health conditions, track falls retrospectively, and predict prospective falls [39].

Traditionally, stationary laboratory equipment, including motion capture systems and force plates, has been used to derive COM- and COP-based balance biomarkers, respectively, for quantifying the dynamics of standing balance. Stationary equipment provides gold-standard reference measurements of COM- and COP-based balance biomarkers for both able-bodied individuals and those with neuromuscular impairment. However, the use of stationary equipment for clinical research and practice can be cumbersome due to the requirement of costly instrumentation in a dedicated lab space, which is not available at many hospitals and rehabilitation clinics [39]. This also results in the limited utility of stationary equipment outside the laboratory environment, compromising the ability of in-home monitoring, performing point-of-care clinical tests [23,41], and routine objective outcome evaluation in clinical research and practice [42]. Wearable IMUs composed of a tri-axial accelerometer, gyroscope, and magnetometer have been used as a reliable alternative for obtaining accurate and sensitive measures of standing balance, given proper positioning and calibration, in different populations, such as able-bodied individuals [43] and populations with Parkinson’s disease [26], traumatic brain injury [23], and SCI [27]. IMUs are lightweight, unobtrusive, and relatively inexpensive. They can be easily integrated into functional tests, making them an ideal alternative to in-lab equipment with high clinical utility [23,44].

Increased use of COM- and COP-derived measures for assessing standing balance performance requires understanding what aspect of postural control these measures quantify and how they can be interpreted. Previous studies have shown that COM motion is controlled by the central nervous system (CNS) via synergistically activating muscles [39,45,46,47], implying that any movements of the COM lead to the initiation of the postural response. Therefore, COM motions indicate challenges in maintaining balance as well as the success of the neural response generated by the CNS to regulate stability [39]. At the same time, COP provides information about the CNS response to COM imbalance, reflecting neuromuscular attempts to respond to instability [39]. Therefore, COP not only contains valuable information about the COM fluctuations but also about the balance control strategy utilized by the CNS [48,49]. Although COM- and COP-based measures may provide overlapping information about postural stability, there is a subtle distinction in what aspects of postural control COM and COP quantify. Mancini et al. [35] showed a weak-to-moderate correlation between COM- and COP-based measures, highlighting the distinction between these measures in quantifying postural control. Therefore, understanding such differences can help researchers and clinicians, particularly physiotherapists, interpret changes in the balance performance of patients and evaluate the outcome of rehabilitation interventions. Utilizing measures of postural steadiness based on COP and COM time series during quiet standing is known as posturography. The following section will review the literature on the two common measurement methods for posturography.

## 3. Posturography

### 3.1. Posturography Using In-Lab Equipment

Previous studies have used COP-based measures to characterize the control mechanisms of quiet standing [33,34,50,51,52]. The COP displacement can quantify standing balance during both dynamic and static conditions [48]. On the one hand, applying external perturbations (e.g., platform translation or external forces) and sensory disturbances (e.g., visual and/or proprioceptive) have been used to characterize, via COP measures, postural responses and underlying stabilization mechanisms during dynamic conditions [45,46,53]. On the other hand, COP displacement during static conditions corresponds to spontaneous body sway and postural control during quiet stance [52]. A commonly applied technique for assessing postural control and stability during quiet standing is utilizing measures of postural steadiness [48]. The most common posturographic measures used to characterize quiet standing include time- and frequency-domain measures, which quantify the displacement, velocity, area, and frequency properties of the COP or COM time-series in the anteroposterior and mediolateral directions [34]. Other measures are stabilogram diffusion function [33,50,51], detrended fluctuation analysis [54], approximate entropy and sample entropy [55,56,57], and Lyapunov exponent [58].

Collins and Luca [33] introduced a new concept for studying human postural control in 1993. They assumed maintaining an erect posture during an upright stance can be viewed as, in part, a stochastic process, and the COP can be analyzed as one- and two-dimensional random walks. They modelled COP trajectories as Brownian motion and assumed that two short- and long-term mechanisms regulate quiet standing. They introduced averaged stabilogram diffusion plots as the mean–square displacement of the COP versus time interval. They estimated short- and long-term Hurst exponents as the slope of the lines that were fitted to the short- and long-term regions of the log–log stabilogram diffusion plots, respectively. The short- and long-term regions ranged from 0 to 0.5 and 2 to 10 s, respectively. They suggested that the Hurst exponent of the short-term interval represents the open-loop mechanism of postural control, while the long-term interval represents the closed-loop mechanism associated with neural feedback over a longer time. Note that the Hurst exponent is a number between 0 and 1, representing how past increments of the COP displacement are correlated with future increments. A Hurst exponent of 0.5 corresponds to zero correlation representing random walk behaviour, whereas a Hurst exponent larger (smaller) than 0.5 corresponds to a positively (negatively) correlated stochastic process representing persistent (anti-persistent) behaviour.

In 1995, Collins and Luca [50] also investigated the effect of visual input on standing balance using the same approach. They showed that visual inputs affect postural control in two ways: a decrease in the stochastic process of the open-loop mechanism or an increase in the stochastic activity of the closed-loop mechanism. They suggested that, in both scenarios, the visual input led to reduced stiffness of the musculoskeletal system. They attributed this observation to reduced muscular activity at lower limb joints for the open-loop mechanism or to reduced gains of proprioceptive and vestibular components of the neural feedback for the closed-loop mechanism. 

**Figure 2:**
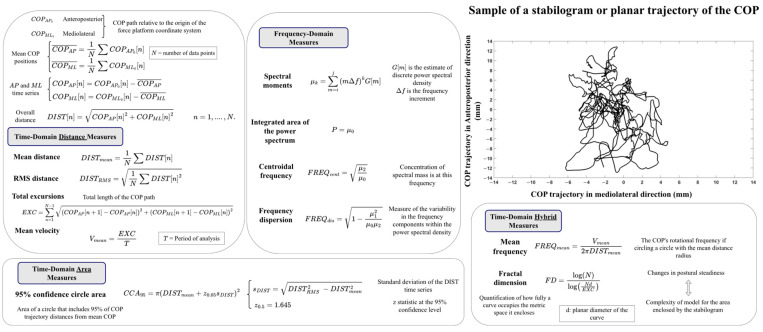
Sample stabilogram of a healthy young adult during quiet standing with eyes open, featuring postural steadiness measures in the time-domain, frequency-domain, and hybrid, as introduced by ref. [34].

Prieto et al. [34] introduced a wide range of measures of postural steadiness based on the planar trajectory of the COP throughout the standing task, called stabilogram, in 1996. These measures were time-domain distance and area measures, frequency-domain measures, and hybrid measures (e.g., fractal dimension). The time-domain measures were associated with the displacement or the velocity of the COP trajectory as well as the area of the stabilogram. The frequency-domain measures characterized the area or the shape of the COP trace’s power spectral density. Hybrid measures modelled the stabilogram with a combination of time-domain distance measures. Figure 2 displays a sample of the stabilogram while showcasing some of the measures introduced in this study. The authors concluded that, among many COP-based measures introduced in this study, the use of (a) one time-domain distance or area measures (e.g., root–mean–square distance), (b) mean velocity, (c) one hybrid measure (e.g., mean frequency), and (d) one frequency-domain measure (e.g., centroid frequency) are recommended to be used together to quantify different aspects of postural control during quiet standing.

COP-based measures characterize postural stability in three domains: stability performance, control demand, and postural regulation. Time-domain distance and area measures are related to stability performance, whereas the velocity measures are related to the control demand [59,60,61,62], and the power frequency measures show alteration in preferential postural regulation [63,64]. An increase in control demand can be attributed to increased visual contribution for postural stability [65,66,67] and an increased risk of falling [34]. Postural instability can be inferred from decreased stability performance, shown by increased distance and area measures, as well as increased control demand, shown by increased velocity measures. Hybrid measures combining the distance and velocity measures characterize the relationship between stability performance and control demand [59]. The frequency measures are oftentimes calculated for the range of 0 to 5 Hz. The centroid frequency is associated with the inertia of an inverted pendulum model [48] and the time required for a system to return to its initial position [59]. Frequency dispersion is a measure of variability in frequency content and is associated with active and passive stiffness or rigidity of the inverted pendulum [52].

Popovic et al. [40] introduced a new stability criterion based on the relationship between the COP position and the individual’s postural stability. They identified four stability zones: a high preference zone as the area where the COP is located 99% of the time during quiet standing, a low preference zone as the area where the COP is located 1% of the time during quiet standing, an undesirable zone as the COP area where the individual needs to change their posture to maintain stability, and an unstable zone as the COP area where the individual needs to take a step to maintain stability. Their proposed measure of stability ranges from zero to one. It is equal to zero in the high preference zone, linearly increases in the low preference zone, and is equal to one in undesirable and unstable zones.

Rocchi et al. [68] identified the features of the COP trajectory that were the most sensitive to postural stability performance to eliminate redundancy. They applied thirty-seven posturographic measures used by Prieto et al. [34] to COP time-series in both the anteroposterior and mediolateral directions, as well as COP resultant distance. They conducted a feature selection process using Principal Component Analysis (PCA) and suggested that the COP resultant distance time-series can be characterized by four measures: (a) the size of the path travelled by the COP (e.g., root–mean–square distance), (b) a frequency characteristic (e.g., centroid frequency), (c) principal sway direction showing the relative weight of anteroposterior and mediolateral components of the COP trajectory, and (d) frequency dispersion. They also recommended six measures for quantifying COP trajectory time-series: (a, b) dispersion in the anteroposterior and mediolateral directions using root–mean–square and their contrast, (c, d) mean velocity in the anteroposterior and mediolateral direction, (e) centroid frequency in the anteroposterior direction, and (f) frequency dispersion in the anteroposterior direction. With the introduction of clearly defined measures for postural stability, the accurate modelling of human standing becomes feasible, enabling researchers to better understand and represent the complex dynamics of postural control.

Human quiet standing is oftentimes modelled as a single inverted pendulum rotating about the ankle joint, stabilized primarily by active control of the ankle joint along with passive musculoskeletal stiffness and damping properties [69,70]. Gage et al. [70] investigated the validity of the inverted pendulum model by examining the relationship between COM and COP during a 120 s quiet standing task in able-bodied individuals. The 3D kinematics of 14 body segments were recorded using optoelectronic motion capture cameras while a force plate measured the COP trajectory and GRFs. Their results validated the inverted pendulum model of quiet standing, showing that the difference between COP and COM trajectories was significantly correlated with COM acceleration. They observed temporal and spatial synchronization between each segment’s COM and the whole-body COM, with the segmental COM increasing linearly with the height above the ankle joint. In addition, they showed that the angular displacement of the ankle joint could track the COM motion.

Fok et al. [71] examined the error of the inverted pendulum model associated with the distance between the body’s COM and the ankle joint during natural unrestricted unperturbed standing as well as the error of having the ankle joint angular motion represent the COM angle. They used an optoelectronic motion capture system, and their experimental procedure included quiet standing with eyes open (EO) and eyes closed (EC), voluntary forward/backward sway, and freely moving. The distance change of the COM during EO and EC quiet standing was very small, i.e., close to the accuracy of their motion capture system. The distance change tended to be larger during forward and backward voluntary sway, but it was not significant. Thus, they suggested that the inverted pendulum model is valid for quiet standing and voluntary sway if the inter-joint contribution, i.e., due to hip or knee joint torques, is not of interest. The COM sway angle and ankle joint angle had a moderate positive correlation during EO and EC quiet standing and voluntary sway; however, a significant offset was observed. This implies that the ankle joint angle moderately represented the temporal features of the COM sway angle, and it may not provide an accurate estimate of COM spatial features.

Aramaki et al. [30] pointed out that hip joint motion also plays a significant role in the efficient maintenance of the COM above the BOS since the literature had suggested that a restricted hip joint led to considerably higher ankle sway in able-bodied individuals [72]. They investigated how the coordination between the ankle and hip joints is controlled during a 30 s quiet standing task with EO and EC. The angular motion of the ankle and hip joints was measured via laser displacement sensors. They observed a significantly higher magnitude of angular position, velocity, and acceleration for the hip joint compared to the ankle joint, confirming that hip joint motion cannot be ignored during quiet standing. Furthermore, they discovered a reciprocal relationship between the angular accelerations of the hip and ankle joints, demonstrating that the ankle angular acceleration was compensated for by hip angular acceleration in the opposite direction. Such a consistent relationship was not observed for the angular displacement. Thus, they suggested that the angular motions of the ankle and hip joints are not used to minimize the COM displacement but, rather, to minimize the COM acceleration.

The ankle and hip strategies, along with stepping, are considered three coordinative patterns employed by the CNS to maintain stability during quiet standing [73,74]. It is a common assumption that quiet stance can be modelled by an inverted pendulum representing the ankle strategy, and the hip joint only contributes during larger perturbations. The ankle strategy predominates during low-amplitude, low-velocity perturbations, whereas the hip strategy predominates during higher perturbation frequencies [73]. However, Creath et al. [74] demonstrated that a single segment model of quiet standing could be inadequate, and both ankle and hip strategies could be observable even during quiet stance. They examined the trunk-leg coordination of able-bodied individuals while standing on a hard surface, foam surface, and a sway-referenced surface with EC. During the sway-referenced trial, the platform was rotated in the anteroposterior direction equal to the hip angular displacement measured via a rod potentiometer. They used spectral analysis on trunk and leg angular displacement under different sensory conditions and observed the angular displacement of the trunk and leg were in-phase at sway frequencies below 1 Hz and anti-phase at sway frequencies above 1 Hz, representing ankle and hip strategies, respectively. The transition from the ankle strategy to hip strategy was abrupt for the hard-surface and foam-surface conditions, whereas a gradual transition was observed for the sway-referenced condition. They suggested that ankle and hip strategies are “simultaneously co-existing excitable modes” that are both present; however, the predomination of one strategy depends on sensory information and the characteristics of the task or perturbation. Similar results were observed by Zhang et al. [75] in both the anteroposterior and mediolateral sway directions, further highlighting the utility of a double-linked inverted pendulum model of the dynamics of quiet standing.

Hsu et al. [32] also proposed that postural control during quiet standing depends on the coordination of multiple joints. They hypothesized that all major joints are equally active along the longitudinal axis of the body and are coordinated to stabilize the spatial positions of the body’s COM and the head. They examined this hypothesis by recording the motion of a multi-segment body model of able-bodied individuals during a five-minute quiet stance task with EO or EC. They showed that the CNS minimizes the sway of the body’s COM and the head during quiet standing by coordinating the variance of joint motions. This coordination ensures that the temporal variability in these joint movements has a minimal impact on either the body’s COM or the position of the head. In contrast to using a multi-segment model, in an inverted pendulum model, the variability of the ankle joint motion would directly lead to variability in the body’s COM and head positions. Depriving vision led to increased joint configuration variance compared to the EO condition, whereas its effect was insignificant on the variation of the body’s COM position, further highlighting the importance of multi-joint coordination in postural control during quiet standing.

Sasagawa et al. [76] investigated the effect of hip joint motion on the body kinematics in the sagittal plane during quiet standing of able-bodied individuals. They derived the actual COM acceleration by dividing the shear force measured by the force plate by the body mass. The estimation of COM acceleration relied on the angular motion data obtained through laser sensors. An anti-phase modulation between the ankle and hip angular acceleration was observed. It was demonstrated that the COM acceleration could be precisely estimated only when both hip joint motion and ankle joint motion were considered.

Hay et al. [77] used magnitude squared coherence (MSC) to investigate the relationship between the free moment and COP in the anteroposterior and mediolateral directions measured by a force plate during quiet standing with EO or EC. Regardless of vision condition, they observed a strong (weak) coherence between the free moment and COP in the anteroposterior (mediolateral) directions at frequencies below 0.5 Hz, whereas the coherence decreased (increased) from 0.5 Hz to 1 Hz. The authors compared their results with previous studies that investigated multi-joint coordination [74] as well as ankle joint and muscle activation coherence [78]. They concluded that these observations resulted from the use of an ankle strategy at lower sway frequencies and the use of a hip strategy at higher sway frequencies.

### 3.2. IMU-Based Posturography

Posturographic measures using gold-standard force platforms and motion capture systems have shown high sensitivity and reliability for characterizing postural control. However, their cost and portability issues have hindered their utilization in clinics. Clinical rating scales are the most common approach for evaluating postural control in clinics; however, they are affected by clinicians’ bias, low sensitivity to mild impairments, and poor reliability [35,79]. Such limitations directly impact the ability of clinicians, physiotherapists, and researchers to identify individuals with mild balance impairment, monitor the progression of the disease, and evaluate the outcomes of interventions [23]. Mancini et al. [35] argued that there is a need for a more practical, objective balance assessment methodology for clinical applications that demonstrates high sensitivity to mild neurological impairments and good test–retest reliability with experimental and clinical validity. Many studies, starting as early as 2002, have proposed IMUs, particularly accelerometers, as a low-cost portable alternative [80,81,82,83]. A waist-mounted accelerometer has been suggested for the approximate estimation of COM motion. In 1995, Winter suggested that COM horizontal acceleration is proportional to the difference between COP and COM and, therefore, could potentially be a better postural sway measure representing the error signal within the postural control system [38].

Whitney et al. [81] investigated the test–retest reliability of three posturographic measures obtained from a waist-mounted accelerometer compared to COP during quiet standing with different sensory conditions. COM acceleration-based measures showed similar test–retest reliability as COP-based measures. Acceleration- and COP-based measures were significantly correlated under all test conditions. They suggested that the use of an accelerometer for balance evaluation may be useful in reducing clinical evaluation time.

Mancini et al. [35] investigated the sensitivity and experimental concurrent validity of using a waist-mounted accelerometer compared to a force plate for measuring postural sway. They also examined the test–retest reliability and concurrent validity of acceleration-based measures compared to clinical scores (postural instability and gait disability sub-score of the Unified Parkinson’s Disease Rating Scale). They used common posturographic time- and frequency-domain measures based on both the COM acceleration and COP time series. They also introduced an acceleration based-measure called JERK [84]. They observed the capability of acceleration-based measures in distinguishing postural sway characteristics of able-bodied individuals compared to individuals with untreated Parkinson’s disease. JERK and the time-domain measures showed high test–retest reliability. They observed a significant correlation between acceleration-based measures and clinical scores. Hence, they suggested that the use of COM acceleration-based measures, including JERK, root–mean–square amplitude, mean velocity, and centroid frequency, are valid, sensitive, and reliable measures of postural stability.

Alberts et al. [85] used an iPad’s IMU composed of accelerometers and gyroscopes attached to the sacrum to approximate the COM and quantify postural stability compared to a force platform in able-bodied individuals. Participants completed a Sensory Organization Test. They calculated the COM sway angle using both IMU reading and COP measured by the force plate. The authors demonstrated that using IMUs could quantify postural stability with sufficient precision and accuracy, as evidenced by the mean absolute percentage error between COG sway measured by the iPad and the Sensory Organization Test. They suggested that using IMUs, at the time, did not replace biomechanical analyses with gold-standard technology that can analyze sensory inputs to differentiate individual contributions of somatosensory, vestibular, and visual factors to postural stability. However, IMUs can translate complex in-lab biomechanical analyses to a broader field, such as clinical evaluations and athletic training.

Heebner et al. [86] investigated the capability and reliability of accelerometry measures, comparing them to COP-based measures, by placing an accelerometer on the lower back of participants. They used these measures to characterize postural stability in healthy athletic individuals during tasks with varying difficulty, including four static tasks with EO or EC and two dynamic tasks. The COM acceleration-based measures showed high reliability, with the ability to differentiate between tasks with varying difficulty. Moderate-to-weak correlation between the acceleration- and COP-based measures, proposed by Heebner et al., was observed, highlighting the fact that the two methods do not represent the same components of postural stability. However, both methods showed similar patterns of postural stability scores across different tasks.

Hansson and Tornberg [87] examined the correlation between a waist-mounted IMU and a force plate, as well as the reliability of the IMU for quantifying standing balance with EO or EC. A strong correlation was observed between two measuring devices in both the anteroposterior and mediolateral directions with EO and EC. They observed moderate to good intra- and inter-trial reliability for the IMU. They suggested that the two assessment devices were not interchangeable, and that there is a significant need for a reliable algorithm with the ability to move the balance assessment using wearable technology out into real life. Moller [88] later developed a mobile application with the goal of preventing falls, called Snubblometer, which operates based on Inertial Measurement Units (IMUs). This application includes a fall risk index, as well as fall and near-fall detection capability. The IMU was placed on one thigh, 10 cm above the knee. Eight different conditions, defined based on the measurement conditions of the IMUs, were evaluated for sensitivity and specificity. The most suitable condition was identified when the system exhibited high sensitivity and specificity in detecting near falls. In this condition, the IMU identified instances where a step significantly deviated from the previous one while also detecting very low acceleration towards the ground.

Ghislieri et al. [89] reviewed the use of wearable IMUs for assessing standing balance. They investigated the application of IMUs for quantifying balance in different populations, sensor placement, common IMU-derived parameters, and validation against the gold standard. Many studies utilized IMUs to assess balance in healthy, young, and/or older adults and individuals with Parkinson’s disease [90,91,92,93] or multiple sclerosis [94,95]. Regarding sensor placement, the most common place was the lower back (e.g., sacral region) as a representation of the body’s COM. Other studies have placed the IMU on areas of the lower limbs, such as the thigh or shank, sternum, upper back, thoracic region, wrist, or forehead. Placing sensors on both the lower back and lower limb enables assessing postural control strategies (e.g., ankle or hip strategies) [90,91]. The most common parameters were acceleration-based measures, including root–mean–square of the acceleration, JERK, range of acceleration, and centroid frequency. Few studies used gyroscope readings. Generally, there was a lack of sensor calibration procedures prior to calculating parameters because it was assumed that sensor misalignment was negligible. Many studies investigated the sensitivity and experimental validity of acceleration-based measures compared to COP-based measures and clinical scores. IMU-derived acceleration-based measures and COP-based measures quantify different aspects of postural control, hindering direct comparison between the two methods. Nevertheless, the authors stated a lack of information about the sensitivity of wearable systems compared to traditional force plate posturography, particularly in the clinical field, for detecting mild changes in balance performance. They highlighted that IMUs had not become a practical tool of posturography for balance assessment due to a lack of accuracy validation of IMU-based measures compared to gold-standard force platforms. Thus, an innovative wearable balance assessment technology can be highly beneficial to both healthy and pathological populations.

Noamani et al. [43] investigated the accuracy of IMUs against the gold standard in-lab equipment (motion capture system) for characterizing standing balance. They used four IMUs placed over the foot, tibia, sacrum, and sternum. The inter-segmental moment and COP were estimated during a two-minute standing task of participants on the force plate using inverse dynamics. They evaluated the implementation of the IMUs’ accelerometer data, both with and without gyroscopes, against a reference method, employing cameras, a force plate, and a bottom-up inverse dynamics approach. They observed that using only 3D accelerometers and a multi-segment model of the human body, we can obtain the joint angles, joint moments, and COP excursion in both the anteroposterior and mediolateral directions during quiet standing with high accuracy. There was no significant difference between results obtained using accelerometer and gyroscope and those obtained using only accelerometers. They concluded that COM- and COP-based balance measures during quiet standing obtained by accelerometers were accurate.

Baker et al. [96] investigated the concurrent and discriminant validity as well as the inter-sensor and test–retest reliability of wearable IMUs for assessing standing balance in healthy adults. The concurrent validity was investigated by comparing IMU-based measures with those of a force plate [35,86] and a combination of motion capture and a force plate [97,98,99], showing moderate to strong correlation in both the anteroposterior and mediolateral directions. The authors observed consistent moderate-to-excellent test–retest reliability for static balance assessment using wearable sensors across different studies. Moreover, discriminant validity was assessed across different studies, highlighting the capability of wearable sensors to distinguish between the balance performance of young and older adults and fallers and non-fallers [100]. Furthermore, a single sensor placed near the body’s COM was as reliable as multiple sensors, showing moderate to good validity and test–retest reliability [96]. It was concluded that using a single waist-mounted accelerometer (over the lumbar region L3–L5) provides simplicity, encouraging clinicians to integrate wearable sensors into practice. This is especially important during telehealth interactions when healthcare is provided remotely. Such a simple wearable device would also increase flexibility for clinical treatment when physical distancing is warranted, such as during the COVID-19 pandemic [96].

Richmond et al. [39] explored current and future applications of COM- and COP-based measures for assessing standing balance. The authors pointed out that traditional posturography using motion capture cameras and force platforms for measuring the COM motion and COP, respectively, could be costly, with reduced utility outside of the laboratory. Recent developments of wearable IMUs and innovative algorithms that can extract useful measures for quantifying standing balance look promising. However, such portable devices are not without their limitations, including sub-optimal noise minimization due to the poor design of internal components and synchronization between sensors [101]. The authors believed that such limitations are being eliminated by recent advancements in the field, and the more prevalent concern is how the outcomes of wearable technologies, in terms of their units, can relate to gold-standard laboratory equipment in practice. Since IMU-based measures have different units of measurement (e.g., angles for orientation) compared to measures obtained from laboratory equipment, such as force plates measuring force and moments, the direct comparison of outcome measures across the literature is cumbersome. Such difficulty can affect the translatability of IMU-based measures to clinical applications. Moreover, the accuracy of IMU-based measures significantly depends on sensor placement and post-processing algorithms. Similar to Ghislieri et al. [89], the authors of this study also believed that there is a major need for a validation study between IMUs and motion capture cameras for measuring the COM kinematics during quiet standing. Given the differences between COM- and COP-based measures, additional investigation is needed to understand the clinical applicability of IMU-derived COM measures compared to traditional COP measures.

**Figure 3:**
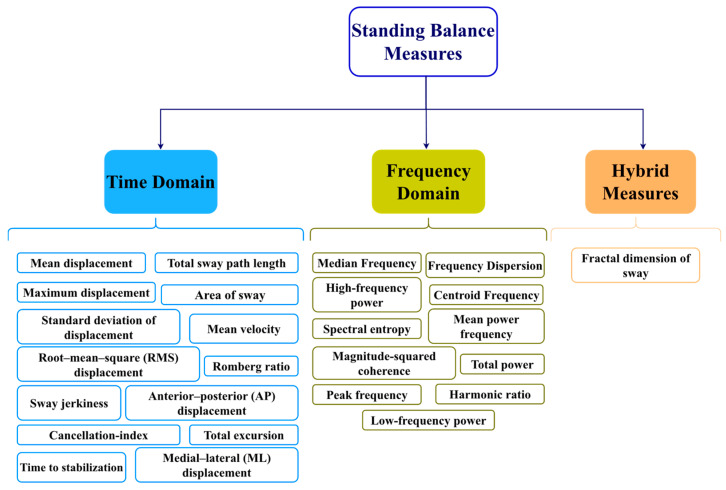
Commonly used standing balance measures based on Center of Pressure (COP) and Center of Mass (COM) (it should be noted that this list is not exhaustive).

Drawing from our analysis of the referenced literature, Figure 3 offers a concise representation of the most commonly used balance measures.

In our review of various studies utilizing IMUs for static balance assessment, a recurring pattern emerged. Most of these studies employed a single IMU positioned at the waist as a representation of the body’s Center of Mass. It should be noted that the IMU placement and whether it can be effectively used on the waist or any other location of the body for assessing balance and movement is decided based on the research question. The suitability of IMU placements for specific clinical populations may also depend on the goals of the clinical assessment and the individual’s unique circumstances. Further research and clinical evaluation would be needed to determine the feasibility and effectiveness of IMU placement in such cases. The investigations covered diverse aspects related to IMUs for balance assessment, including their test–retest reliability, the correlation between IMU-derived measures and those obtained from force plates, a combination of force plates and motion capture, as well as traditional clinical assessment scores. There have also been recent efforts to investigate the accuracy of IMUs against the gold standard in-lab equipment for static balance assessment. The collective findings of these studies indicate that IMUs offer relatively high sensitivity and reliability. They can be effectively employed in clinical evaluations, particularly in settings where access to complex and expensive in-laboratory equipment is limited or unavailable.

In conclusion, in the context of in-lab balance assessments, sophisticated tools such as motion capture cameras, force plates, and laser displacement sensors are employed to precisely measure multiple body segments. These technologies yield accurate data regarding the body’s Center of Mass (COM) and the trajectory of the Center of Pressure (COP). In this context, balance markers often emphasize the time and frequency aspects of the COP trajectory. In contrast, most IMU-based studies rely on approximating COM acceleration and examining various measures defined based on this parameter. As a result, IMU-derived acceleration-based measurements and COP-based measurements capture different aspects of postural control, making direct comparisons between the two methods challenging. Although IMU-based markers may not capture all the information regarding participants’ balance performance, as mentioned earlier, they have proven useful in distinguishing various scenarios and participants with differing levels of static balance capabilities.

Having conducted an extensive review of in-lab and IMU-based posturography, the focus now shifts towards a comprehensive examination of balance assessment in older adult population and individuals with incomplete spinal cord injury (iSCI). The rationale behind selecting these specific populations lies in their significant prevalence, making them pertinent populations for investigation. By exploring balance assessment in these distinct groups, the aim is to uncover the unique aspects and challenges each demographic encounters due to their individual physiological and neurological characteristics. While age-related changes, such as sensory deficits and muscular weaknesses, can impact balance in older adults, individuals with iSCI face impairments resulting from damage to the spinal cord, which also leads to altered sensorimotor function. Thus, studying balance in these populations aligns with the broader objective of developing personalized interventions to enhance standing balance and reduce fall risk for these vulnerable groups. Through an in-depth review of the methodologies, techniques, and advancements tailored to these specific populations, we aim to advance our understanding of postural control and contribute valuable insights to the field of balance assessment, ultimately striving to improve the overall well-being and quality of life for older adults and individuals with iSCI.

## 4. Balance Assessment in Older Adults

As was highlighted earlier, falls are notably prevalent among older adult population [6,7]. Therefore, it is not surprising that falls are among the most common causes of injuries in senior adults [1,2]. Poor postural stability is a key contributor to falls in seniors, and balance evaluation is an effective measure for introducing targeted rehabilitative interventions [102], which can prevent more than 50% of potential falls in seniors [103]. Targeted rehabilitative interventions are carried out to not only prevent future falls but also reduce fall severity [8]. In this light, to reduce future fall incidences and their adverse consequences among senior adults, it is essential to (1) implement effective balance assessment methodologies, (2) introduce targeted patient-specific rehabilitative interventions, and (3) evaluate the effectiveness of such interventions [1,8,104].

The BBS test is also commonly used in geriatric clinics for assessing the balance performance of older adults and for the clinical outcome evaluation of rehabilitative interventions. It was previously discussed that, although BBS is relatively fast and reliable, it tends to be, in part, subjective in nature due to the involvement of human decision-making [105] and low construct validity and may not always result in reliable and sensitive outcomes [26]. In addition, clinical scales provide little information for understanding potential underlying causes of balance difficulties and for evaluating the effect of therapy on balance performance [16,21,22]. Hence, there is a need for a more quantitative, objective methodology to evaluate balance when choosing targeted rehabilitative interventions and when performing their objective outcome evaluation [35,79]. The literature has shown that quantitative balance biomarkers such as COM- and COP-based measures allow for identifying age-related changes in balance and, thus, the risk of falling in the older adult population with high sensitivity and reliability [34,41,106,107,108].

Collins et al. [51] used the stabilogram diffusion function to investigate age-related changes in characteristics of open- and closed-loop control mechanisms of balance. They measured the COP trajectories using a force platform during quiet standing in young and senior adults. They obtained short- and long-term diffusion coefficients and Hurst exponents as the slopes of the lines fitted to the short- and long-term regions of the linear–linear and log–log stabilogram diffusion plots, respectively. They observed that the steady-state behaviour of the open-loop postural control in senior adults was more positively correlated and, thus, more unstable compared to young adults. At the same time, they observed that the steady-state of the closed-loop postural control was more negatively correlated in senior adults, implying a more stable closed-loop mechanism over the long term. Interestingly, seniors used the open-loop control mechanism for longer time intervals, which could be associated with a larger COP displacement in this population. This suggests a longer delay in utilizing the closed-loop control mechanism in senior adults.

Panzer et al. [109] performed a biomechanical assessment of quiet standing to investigate age-related changes in postural stability. They collected kinematic and kinetic data using motion capture system and force plate, respectively. They used the mean, variability, and total path length of the body’s COM, individual body segments, and COP. They observed a significant positive association between aging and the variability of COM, head, and hip motions. They observed an altered postural control strategy in older adults compared to young adults as a compensatory strategy for primary balance deficits. Older adults showed reduced small continuous movements while exhibiting larger adjustments involving the trunk and hip motions. They also observed an increased COP path length in the anteroposterior direction during the EC condition without any significant change in the COM path length. Based on this result, the authors believed there was no evidence of reduced postural stability concurrent with aging since balance maintains the COM over the BOS, and therefore, the assessment of balance must be based on the COM. However, they stated that postural adjustments due to aging could be less effective when balance is challenged.

Prieto et al. [34] compared a variety of COP-based time- and frequency-domain measures using a force plate to investigate age-related changes in postural steadiness during quiet standing with EO or EC. They observed that different measures could distinguish EO and EC conditions in young and senior adults. The mean velocity of COP was the only measure that could concurrently identify age-related changes and eye conditions in balance. They suggested that multiple measures are required to adequately characterize changes in balance due to aging as well as both eyes conditions within each age group. They recommended the use of COP mean velocity; one time-domain distance measure, such as root–mean–square distance; one time-domain hybrid measure, such as mean frequency; and one frequency-domain measure, such as centroid frequency, as they may characterize different aspects of postural stability.

Freitas et al. [110] investigated the effect of aging on human postural control during prolonged standing. Young and senior adults performed 30 min and 60 s quiet standing trials on a force plate. Both groups showed an increased COP root–mean–square distance and velocity during prolonged standing. However, the older adult group exhibited smaller changes in postural sway during prolonged standing. The authors attributed this to the lack of mobility in older adults, which may contribute to an increased risk of falling in this population.

Raymakers et al. [111] investigated the choice of COP-based measures for assessing standing balance with EO or EC on both a hard surface and foam surface with or without cognitive tasks using a force plate for able-bodied young and older adults and older adults with balance impairments. They used COP-based measures, including mean velocity, maximal anteroposterior and mediolateral range of movement, area, deviation, and the stabilogram diffusion function. Mean velocity was the most consistent measure, showing differences between test conditions, health conditions, and age; however, it was not discriminative when the cognitive task was introduced to able-bodied older individuals. Maximal mediolateral movement range was discriminative among groups and test conditions, but not the cognitive task in able-bodied young and older adult individuals. The critical time derived from the stabilogram diffusion plot exhibited no association with other measures while distinguishing the effect of the cognitive task in older individuals standing on the hard surface. They concluded that the mean velocity seemed to be the most informative measure of balance in all groups and conditions.

Amoud et al. [54] used fractal time-series analysis to quantify postural stability of older adults compared to able-bodied young adults using a force plate. They employed two methods for computing the Hurst exponent for fractal and nonlinear time series analysis using COP time series: detrended fluctuation analysis and stabilogram diffusion analysis. They used three different sizes of sliding windows (2.5, 5, and 10 s) and investigated the effect of age, sliding window time, and computation method on the Hurst exponent, while ICC was used as a reliability measure. Both methods were able to identify age-related differences in postural stability with the sliding window time of five seconds, with good-to-excellent ICC for the detrended fluctuation analysis method showing more robustness.

When using the inverted-pendulum model of standing balance, the distance between the COP and vertical projection of COM (COP–COM) shows the relationship between the controlling and controlled variables of postural control. The COM acceleration is proportional to COP–COM. Masani et al. [112] claimed that, since aging affects postural control, these two variables could be affected. They compared the COP–COM and COM acceleration between a group of able-bodied young adults and older adults during quiet standing with EO or EC. They observed higher COP–COM variability and larger COM acceleration in older adults compared to young adults, irrespective of the vision condition. They observed that COM acceleration was proportional to COP–COM in both groups, implying the validity of using the inverted pendulum model in older adults. They suggested that the increase in variability in COP–COM due to aging resulted from changes in the postural control strategy and, consequently, COM acceleration became larger.

Lin et al. [113] investigated the within-day and between-day reliability of COP-based measures for identifying age-related differences using a force plate. COP was recorded during quiet standing on four different days. The COP-based measures included root–mean–square distance, mean velocity, median frequency, sway area, and detrended fluctuation analysis. The standard error of measurement and ICC were used to quantify reliability. Within-day reliability was higher than between-day reliability. Consistent with previous studies, mean velocity was the most reliable measure. Older participants, compared to younger individuals, exhibited better ICC for all COP-based measures and comparable standard error of measurement, except for mean velocity and sway area.

Tucker et al. [114] investigated age-related differences in postural reaction time and coordination during voluntary sway in the anteroposterior and mediolateral directions in response to an auditory cue initiated from static quiet stance or dynamic switching of sway between two directions. Participants stood on a force plate while wearing tri-axial accelerometers mounted on the head and lower trunk. Reaction time was defined as the difference between cue onset and the first observable change in COP or acceleration. Measures included reaction time, the difference between reaction time of COP, head and trunk acceleration, and COP–head–trunk coupling. The older adult group exhibited a slower reaction time during both static and dynamic tasks. They observed a smaller difference between reaction time and phase between COP, trunk acceleration, and head acceleration. They suggested that the older adult group adopted a more rigid coordination strategy compared to younger adults. This could be a compensatory strategy in response to balance difficulty in older adults compared to younger adults.

O’Sullivan et al. [105] argued that measurement of COM acceleration with a relatively inexpensive, lightweight, body-worn accelerometer could be a potential solution to the subjectivity of clinical scales and a potential alternative to expensive in-lab equipment. They investigated the correlation of accelerometry with the BBS and Timed Up and Go (TUG) tests and characterized the accelerometer response to challenging balance conditions in fallers and non-fallers. Older adult patients of a hospital identified as fallers and non-fallers participated in the study. COM acceleration was measured using a waist-mounted accelerometer during standing with EO or EC on both a hard surface and foam surface. The root–mean–square of COM acceleration increased with task complexity. The accelerometry could distinguish fallers and non-fallers under the foam-surface EO condition. A high negative correlation between the accelerometry and BBS and a positive correlation between the accelerometry and the TUG were observed for the foam-surface EO condition. Therefore, they demonstrated that accelerometry could be an efficient quantitative approach for assessing balance in older adults.

Singh et al. [115] investigated the effect of age, vision, and surface compliance on the spectral content of COP time series. Able-bodied young and older adult individuals stood on a force plate with EO or EC on a hard surface or foam surface. The mean power spectral density of COP over discretized bands was calculated. The effects of vision, surface, and age were distinguishable using this measure in the AP and ML directions. They observed, in older adults, a significant change in the spectral content of the COP in both AP and ML directions with task difficulty (EO vs. EC and hard surface vs. foam surface). They suggested that vision and surface condition were predominantly related to the musculature responses associated with body sway in the anteroposterior and mediolateral directions. The authors suggested that using the spectral content of COP to distinguish the contribution of different sensory inputs to postural control could be useful for identifying older adults individuals with impaired balance. Similar results were observed by Fujimoto et al. [116].

The literature has shown that the anti-phase action between the leg and trunk reduces the COM acceleration during quiet standing, playing a major role in postural stability [30,74]. Kato et al. [37] investigated the effect of aging on trunk–leg movement coordination during standing. They measured trunk and leg motion in the anteroposterior direction using laser displacement sensors in young and older adults participants. They observed significantly higher COM velocity and acceleration in older adults compared to young individuals. They also observed increased angular acceleration of the trunk and leg segments as well as reduced trunk-leg anti-phase action. They concluded that reduced trunk–leg anti-phase action due to aging is the major contributor to increased COM acceleration and, thus, is responsible for reduced postural stability in older adults.

Li et al. [117] assessed the reliability and validity of COP-based measures for balance assessment in older adults. Participants were evaluated using both BBS and a force plate two times a week apart. They used ICC and Pearson correlation to assess the reliability and validity of COP-based measures, respectively. Good-to-excellent reliability was observed for twelve COP-based measures. A moderate-to-good negative correlation was observed between the measures and the BBS. Therefore, the authors recommended the use of COP-based measures as reliable, valid measures for balance evaluation in older adults.

Ghahramani et al. [118] analyzed the postural sway of senior adults categorized as non-fallers, once-fallers, and multiple-fallers using clustering techniques. They performed quiet standing with EO, EC, and feet together as well as single-leg and tandem standing. Trunk angular position and velocity in the frontal and sagittal planes were measured using an IMU attached to the lower back. Data clustering techniques were applied to the recorded data in the form of unsupervised learning to identify meaningful patterns between groups and test conditions. A sway index was defined as the ratio of the difference between the number of all data samples and the number of data in the common cluster over the number of all data samples. The sway index of standing on one foot and with one foot in front of the other could distinguish older fallers from non-fallers with comparable sensitivity and specificity with BBS. Therefore, they suggested that such a protocol could be an alternative or a complementary approach to BBS for identifying older fallers.

A similar study was conducted by Johansson et al. [119]. They investigated how postural sway measures could predict fall incidences in 1900 community-dwelling older adults. Postural sway was measured during quiet standing with EO or EC using a force platform. The COP path length was used as a measure. A TUG test was also carried out to assess lower leg muscle strength, gait performance, and functional mobility. A hydraulic hand dynamometer was used to measure the maximum grip strength of the non-dominant hand. Participants reported incidents of falls six and twelve months after the examination. COP path length was significantly greater among fallers than that of non-fallers during both EO and EC conditions, with a strong correlation between the two trials. The authors obtained a nonlinear distribution of falls and COP path length during the EO condition by dividing the path lengths into quintiles, with significantly increased fall frequency in the fifth quintile. Independent predictors of falls were explored using two logistic regression models. The first classification model used only the COP path length as an independent predictor of falls, whereas the second model included other measures (e.g., sex, weight, grip strength, TUG). They observed that postural sway quantified by COP path length could independently predict fall incidents in community-dwelling older adults, highlighting the importance of posturographic measures as a predictor of future falls in the older adult population.

In a comprehensive study, Pizzigalli et al. [108] reviewed the postural characteristics of older adults with a high risk of falling based on static and dynamic balance assessments. They observed that COP path length, mean velocity, and sway in the anteroposterior and mediolateral directions are the main measures capable of distinguishing fallers and non-fallers in the older adult population. COP–COM was found to be a reliable postural stability measure in healthy older adults, and it can potentially be used to track clinical changes in this population. Common COP-based time- and frequency-domain measures exhibited better ICC and SEM with aging except for sway area and mean velocity. Older adults have shown weaker muscle strength and resistant torque in dynamic conditions compared to younger adults, affecting their ability to restore postural stability. Furthermore, older adults require higher muscle activity compared to younger adults to produce a resistant torque, leading to premature fatigue and increased fall risk. Balance indicators in the mediolateral direction, including COP-based mean velocity, mean amplitude, and root–mean–square displacement during EO and EC conditions, were the best indicators for identifying postural stability differences between future fallers and non-fallers among older adults. Tandem standing and standing with feet placed together have shown to be effective tests for distinguishing fallers from non-fallers. Standing with EC could also be effective since fallers had lower proprioception and more reliance on visual inputs. Standing on a foam surface has shown a higher increase in COP path length in non-fallers compared to fallers, implying that fall-prone older adults seemed to be less affected by the reduction in somatosensory input. Older adults exhibited a reduced cutaneous sensation causing an inability to detect the COP movement under their feet. This inability can delay compensatory reactions when a fall happens, making reaction time one of the best identifiers of fallers. Older adults, compared to younger adults, had more antagonist muscle activation adopting a hip strategy to restore postural stability in dynamic conditions. During voluntary sway, older adults have shown slower, less reliable, and less responsive postural reflexes, suggesting issues with hierarchical movement organization. In addition, reduced muscle strength (e.g., ankle dorsiflexion weakness) due to atrophy, deterioration of mechanical properties, and loss of motor units are responsible for reduced postural stability in older adults. Moreover, greater postural sway and higher muscle activations correlated with lower clinical scores and increased fall risk in older adults. The authors concluded that objective balance evaluation in older adults is essential for fall risk prediction and evaluation of the effectiveness of balance training programs aimed at preventing future falls.

Roman-Liu [106] performed a systematic review and meta-analysis of available data to investigate age-related changes in COP range and velocity in the anteroposterior and mediolateral directions. They formed a numerical database with the mean and standard deviation of selected COP-based measures classified by eye condition (EO or EC) and age group (younger or older adults). Their results showed that body sway range and velocity increased with age, with the velocity measure being more affected by age-related changes. COP measures were higher for the EC condition and for older adults, and thus, quiet standing with EC could provide clearer results for identifying age-related changes. They concluded that such quantitative measures of stability with cut-off scores could be used for generating standards and recommendations for balance evaluations.

Montesinos et al. [120] reviewed the application of wearable IMUs for fall risk evaluation in older adults. They identified the optimal combination of sensor placement, task, and feature categories for assessing the risk of falls. They employed a three-way contingency table with the three mentioned covariates, using only features where the mean value significantly differed between fallers and non-fallers. Their results showed that (1) angular velocity during walking measured via an IMU attached to the shank and (2) linear acceleration during quiet standing, sit-to-stand, and stand-to-sit measured via an IMU attached to the lower back are the best combination. The measures that were significantly higher in older adults fallers were (1) the root–mean–square acceleration in the mediolateral direction during quiet standing with EC, (2) the number of steps and total time to complete a TUG test, and (3) step time during walking.

Sun and Sosnoff [121] reviewed the sensing technologies used to provide objective fall risk assessment in older adults. Four major sensing technologies were IMUs, video/depth cameras, pressure sensing platforms, and displacement laser sensing. Assessment tasks included walking, static/dynamic balance, and functional mobility. The authors believed that the variation in outcome measures, sensor location, task, assessment tools, and modelling techniques hinder any conclusion on the capability of such technology in predicting future falls in older adults. The outcome variables in the assessment of standing balance using IMUs included parameters such as RMS acceleration of COM and frequency variability, while studies employing depth cameras reported measures such as COM sway area and sway velocity. Studies that employed pressure-sensing insoles measured parameters such as COP path and temporal gait parameters in the test for straight-line walking. IMU sensors were positioned on various body parts, including the lower back, thigh, head, and foot, among others. The tasks performed in these studies encompassed activities such as assessing standing balance and conducting clinical tests such as the BBS and TUG, as well as straight-line walking. A wide array of modelling techniques, including linear regression, Support Vector Machine, Naive Bayesian classifier, Decision Tree, Cluster analysis, and k-Nearest Neighbour were employed across different studies to predict fall risk. The authors concluded that there is a need for appropriate model construction and validation before using such technologies for fall risk assessment in everyday life. In addition, most previous studies have used a retrospective fall history along with clinical scales such as BBS and TUG as a reference for identifying fall risk, which may not be accurate. The authors suggested the use of prospective fall occurrence at six months post-examination as a better alternative. Nevertheless, future work is needed to identify clinically meaningful and easy-to-interpret outcome measures for identifying fall risk in older adults based on evident research.

Patel et al. [100] investigated gait and posture differences between older adult fallers and non-fallers based on the measurements of wearable IMUs. They identified 149 characteristic gait and posture differences. Spatiotemporal measures, including slower walking speed, shorter step and stride lengths, as well as acceleration-based measures such as reduced root–mean–square acceleration, were the measures highly attributed to the risk of falling when performing dynamic tasks. At the same time, increased root–mean–square acceleration of the trunk was an indicator of fall risk during static tasks. A single waist-mounted IMU was a successful choice for determining various gait and posture characteristics. However, there was a lack of studies conducted outside of laboratories such as clinics. IMUs are a promising tool to be integrated into current clinical fall risk evaluation methods, and they are even more effective for continuous unsupervised monitoring. Due to the portable, lightweight nature of IMUs, they can be used outside of a laboratory environment, which has not been widely investigated. The authors suggested that the use of IMUs allows clinicians to make a more objective, informed assessment of fall risk and, therefore, their application in a clinical setting should be further investigated in the future.

In a clinical study, Noamani et al. [122] investigated the integration of wearable sensors into observational clinical tests for objective outcome evaluation of balance rehabilitation in older adult fallers. They used IMUs placed at the sternum, sacrum, and tibia of older adult fallers for objective balance assessment during the BBS test in in-patient older adults fallers in a geriatric clinic. The calculated objective balance biomarkers, such as COP time- and frequency-domain measures, COM acceleration time-domain measures, and intersegment coordination measures, were able to distinguish older adult fallers from able-bodied young adults. Also, balance biomarkers of older adults were able to identify the effect of rehabilitative intervention, exhibiting a reduced sway acceleration and jerkiness in the medial–lateral direction post-rehabilitation. The findings demonstrated the potential for using instrumented balance assessment to obtain quantitative and objective measurements for clinical evaluations.

## 5. Balance Assessment in Individuals with iSCI

The literature has reported that regaining the ability to maintain postural stability and walks are among the top priorities for individuals with iSCI [16,19,20]. Up to one-third of all individuals with recent SCI are able to regain partial balance and walking ability after the first year post-injury [16,123]. However, their prospective level of ambulation is related to the motor function below the lesion level [124]. For instance, 80–100% of individuals with iSCI rated D on the American Spinal Injury Association Impairment Scale (AIS) are able to partially recover walking function, indicating some preservation of motor and sensory function below the level of lesion one year post-injury [16,125]. This shows the necessity of implementing outcome measures that allow for the identification of balance and walking abilities of individuals with iSCI to design more effective rehabilitative interventions [16].

The integration of sensory information from the visual, vestibular, proprioceptive, and somatosensory systems plays a predominant role in effective postural control [10,17]. While iSCI may result in motor impairment below the level of the injury [18], it may also change sensory reweighting due to, e.g., reduced somatosensation. Developing compensatory strategies to maintain postural stability post-iSCI leads to an alteration in reweighting sensory information [19,21]. As a result, alteration of sensory information, such as visual [17] and somatosensory [126] inputs, can further challenge postural control in individuals with iSCI. Hence, the implementation of methodologies for identifying changes in postural control and underlying impaired balance mechanisms allows for targeted and guided rehabilitation after iSCI [21]. Such interventions can have a positive impact on the improvement of postural control and movement coordination [19].

Sayenko et al. [19] used COP-based measures from a force plate to investigate the effect of the visual feedback on standing balance in individuals with iSCI. They also determined whether static and dynamic stability could be improved during training-irrelevant tasks after balance training. Participants attended 12 training sessions over four weeks. During each training session, they stood on a force plate and were asked to look at a monitor placed at their eye level in front of the force plate. The planar COP position was used as an input for the game-based exercises. In addition, static and dynamic stability was evaluated before and after training. Static balance was evaluated using COP distance, area, and velocity measures obtained during quiet standing with EO or EC. Dynamic balance was evaluated based on the maximum voluntary displacement of COP toward eight targets placed 45 degrees apart around the center without losing balance. The displacements of COP in eight directions formed an octagon, and its area was calculated as a measure of dynamic stability. They observed a significant improvement of static and dynamic stability measures post-training, showing the effectiveness of visual feedback on the postural control of individuals with iSCI. This could be attributed to the improvement of existing motor strategies as well as the development of new strategies and integration of the sensorimotor system.

Grangeon et al. [59] determined the minimum COP-based measures required for characterizing seated stability in individuals with SCI compared to able-bodied individuals by comparing 39 COP-based posturographic measures. Two sitting positions were performed by each participant, first with both hands on the thighs, and second with both upper extremities flexed and abducted at 70 degrees and 45 degrees, respectively. COP-based measures were able to distinguish balance of individuals with SCI compared to able-bodied participants irrespective of sitting position. Bilateral hand support led to reduced anteroposterior sway in individuals with SCI and was suggested as a compensatory strategy. COP time-domain distance and area measures were highly correlated, whereas frequency-domain measures were discriminative and uncorrelated. They suggested that posturographic measures for characterizing balance post-SCI should include mean distance, mean velocity, centroid frequency, median frequency, and frequency dispersion in both the anteroposterior and mediolateral directions.

Lemay et al. [17] compared the performance of individuals with iSCI and able-bodied individuals in terms of the use of visual information to maintain standing balance. They also quantified the relationship between the contribution of visual inputs to postural stability and a clinical balance scale. All participants performed two 45 s quiet standing trials on a force plate with EO or EC. They used root–mean–square distance, mean velocity, and sway area as posturographic measures. Individuals with iSCI were also assessed with Mini-BEST as a clinical balance scale. They observed worse postural stability in individuals with iSCI compared to able-bodied individuals in both conditions. Moreover, the Romberg ratios (i.e., the ratio between the measure in EO and the measure in EC) of mean velocity and sway area were significantly larger for individuals with iSCI compared to able-bodied individuals, implying a higher contribution of visual inputs to postural steadiness post-iSCI. Romberg ratios of root–mean–square distance and sway area were significantly correlated with Mini-BEST. Since iSCI causes somatosensory impairments following a lesion, the contribution of visual information during standing may be increased post-iSCI in comparison with able-bodied individuals due to altered sensory reweighting.

Maintaining balance is an essential component of safe standing and walking; however, it is a major challenge for individuals with iSCI as they regain the ability to walk [17]. Normal walking patterns, higher stride speed, less reliance on assistance, and more functional ambulatory ability are correlated with greater postural control in individuals with iSCI [16]. The literature has demonstrated that clinical measures of standing balance, such as BBS scores, for individuals with iSCI correlate well with various walking outcome measures, such as speed, endurance, and reliance on mobility-related assistive devices [16,22]. However, the major bottlenecks of the BBS are its ceiling effect, its inability to predict future falls, and the inability to determine the underlying cause of balance difficulty [16]. As a complementary approach, Lemay et al. [22] investigated the concurrent validity of Smart Balance Master (NeuroCom International Inc., Clackamas, OR, USA) tests in individuals with iSCI compared to observational BBS. The Smart Balance Master is designed to evaluate a person’s balance, posture, and stability. It consists of two force platforms used to determine the COM position, an eye-level screen showing the participant’s COM, and the evaluation task. In addition to BBS, participants performed a static task, including standing on the force platform with EO and then EC with sway area as the measure. Then, a limit of stability task was performed where participants were asked to reach eight equally spaced targets as fast as possible. Sway path length and time were averaged over eight directions. Finally, participants performed a weight-shifting task in the anteroposterior and mediolateral directions by following a target with their COM shown on the screen, with the absolute error expressed as a percentage of the limit of stability being reported. They observed no stability performance difference between individuals with tetraplegia and those with paraplegia using posturographic measures. The limit of stability test showed the highest correlation with BBS and other Smart Balance Master tests. Therefore, the limit of stability test was suggested as a complementary method to BBS for assessing the dynamics of standing balance in individuals with iSCI.

In another study, Lemay et al. [18] characterized dynamic postural balance during standing among individuals with iSCI compared to able-bodied individuals using the comfortable multi-directional limit of stability and investigated its association with quiet standing posturography. Participants were asked to lean toward eight targets placed 45 degrees apart while standing on a force plate, and COP visual feedback was provided. The absolute maximal distance and the path length of COP were calculated for each direction. Furthermore, quiet standing was performed with EC, and time-domain COP measures were computed. The observed COP path length was significantly greater for individuals with iSCI compared to able-bodied individuals in all directions except for the anteroposterior direction. The maximum position reached in the anteroposterior direction was significantly smaller in individuals with iSCI. They observed little correlation between quiet standing time-domain measures and the limit of dynamic stability. They suggested that a comprehensive assessment of postural stability should also include outcome measures evaluating both static and dynamic stability.

Tamburella et al. [127] analyzed the reliability, validity, and responsiveness of COP-based measures to assess standing balance under different conditions in individuals with iSCI. They examined 23 individuals in 111 sessions over one year. Each session included clinical scale tests, such as BBS and stabilogram analysis on a force plate. Test conditions comprised open feet and closed feet with EO and EC. COP-based measures were path length, mean velocity in the anteroposterior, and mediolateral directions and their resultant ellipse area, x-axis, and y-axis of the ellipse area. Among all COP-based measures, mean velocity was the most repeatable measure, with the lowest coefficient of variation and highest intraclass correlation coefficient (ICC). Path length and mean velocity in the mediolateral direction and the mean velocity resultant had the lowest percentage change due to the measurement error quantified by Minimal Detectable Change. Path length and mean velocity in the anteroposterior and mediolateral directions and their resultant were also the most valid measures with the highest correlation with BBS. Among the test conditions, the open-feet test with EO had the highest criterion validity, whereas the open-feet test with eye closed was the most reliable with the highest ICC. In terms of responsiveness, BBS was the most sensitive clinical scale, whereas all COP-based measures were shown to be more sensitive than all clinical scales.

In a systematic review, Arora et al. [10] explored balance measures used to evaluate the balance performance of individuals with iSCI and compared them in terms of their clinical utility, psychometric properties, and comprehensiveness. They identified 31 balance measures, including eleven biomechanical measures and twenty balance scales. Balance scales have shown higher clinical utility compared to biomechanical measures (e.g., COM- and COP-based measures), limiting the use of biomechanical measures in a clinical environment. Therefore, developing biomechanical measures with high clinical utility is of significance. Among balance scales, the BBS and Functional Reach Test had higher validity [16,128,129,130], reliability [127,130,131], and responsiveness [128,132]. Although BBS was the most common test, it was not able to predict future falls in individuals with iSCI [131,133]. The comprehensiveness of the clinical measures was based on how many domains of postural control they could evaluate, including static stability, underlying motor systems, functional stability limit, verticality, reactive postural control, anticipatory postural control, dynamic stability, sensory integration, and cognitive influences. The Mini-BEST was the most comprehensive among clinical scales. The authors believed there was no single test or measure that concurrently demonstrated high clinical utility, strong psychometric properties, and comprehensiveness. Three gaps were identified by the authors: First, the measures should be further investigated for their psychometric properties in individuals with sub-acute and chronic SCI, with the focus on identifying cut-off scores indicating a high risk of falling. Second, the responsiveness of the measures to changes in postural control in individuals with SCI should be further studied. Third, comprehensive balance measures during transferring in wheelchair users should be further investigated.

Chan et al. [21] evaluated the test–retest reliability of Mini-BEST via ICC and assessed the concurrent validity of Mini-BEST by examining its Pearson’s correlation with COP-based measures. COP-based measures have shown high reliability and validity as the gold standard in the literature [127]. The Mini-BEST evaluates balance via 14 standing and walking tasks scored on a three-point ordinal scale. Participants performed the Mini-BEST twice, two weeks apart. They also performed a quiet standing test on a force plate with EO or EC. They observed excellent test–retest reliability of the Mini-BEST. They observed a negative correlation between the Mini-BEST score and COP mean velocity during the EO condition. In terms of convergent validity, they observed a strong correlation between the Mini-BEST total score and lower extremity strength.

In a prospective cohort study, Musselman et al. [134] investigated the performance of COP-based measures and clinical scores for distinguishing fallers and non-fallers in ambulatory individuals with iSCI. Participants completed two test sessions. In the first session, participants performed quiet standing with EO or EC on a force plate. The mean velocity of COP was calculated in the anteroposterior and mediolateral directions. In the second session, a physical therapist performed a clinical assessment, including lower extremity strength, proprioception, cutaneous sensation, walking speed, and balance self-efficacy. Participants then self-reported their falls for one year after test sessions. Participants were classified as fallers if their number of falls exceeded the median number of falls among all participants. Outcome measures of lower extremity strength, cutaneous pressure sensitivity, walking speed, and the COP mean velocity in the mediolateral direction could distinguish fallers from non-fallers. The authors suggested that COP mean velocity, along with the above-mentioned clinical scores, could be useful for the clinician to identify ambulatory individuals with iSCI with a high risk of future falls.

Many studies mentioned so far have taken advantage of in-lab equipment, such as motion capture cameras and force plates, along with clinical scales to obtain objective measures of standing balance in ambulatory individuals with iSCI. However, the use of wearable IMUs to obtain clinically meaningful measures of standing balance in individuals with iSCI has been rarely addressed and can promote the combination of objective balance assessment and clinical scales of balance in clinical settings. Recently, Noamani et al. [135] used a single waist-mounted IMU to characterize standing balance of individuals with iSCI standing in various challenging conditions, i.e., on a hard surface and foam surface with EO and EC. Comparing the results with an age-matched able-bodied control group, reduced stability performance, an increased control demand, and a less effective active correction post-SCI were observed in all standing conditions. The individuals with iSCI, who experienced impaired somatosensory feedback, showed a higher and lower reliance on visual and somatosensory information, respectively. Therefore, wearable IMUs were able to objectively assess post-iSCI standing balance.

Furthermore, since individuals with iSCI suffer from impaired sensorimotor function and dysfunctional postural control, they may adapt different movement strategies compared to able-bodied individuals to compensate for reduced postural control. COP- and COM-based measures are strong indicators of impaired postural control. However, they cannot directly reveal all aspects of the adaptive postural stability strategies employed during impaired standing [95]. Therefore, identifying alteration of postural control strategies post-iSCI compared to able-bodied individuals under different sensory conditions is a significant need. To address this gap, Noamani et al. [136] assessed the balance control strategy and inter-segment movement coordination of an iSCI population by studying the acceleration patterns recorded by the IMUs placed on the pelvis and shank of the study participants. The tests were conducted with four standing conditions on a hard surface and foam surface with EO and EC. A similar balance strategy at lower frequencies was observed between the iSCI and able-bodied populations. However, a decreased ability to adapt inter-segment coordination between trunk and leg segments, transitioning from ankle strategy to mixed ankle-hip strategy as sway frequency increases, was observed post-iSCI. Furthermore, it was demonstrated that trunk–leg movement coordination in both populations could be affected by alterations in somatosensory inputs, as evidenced by the coherence between trunk and leg accelerations. The utilization of coherence analysis to characterize the coordination of trunk–leg movements demonstrated high sensitivity and reliable discriminative ability in identifying changes in balance control strategy following iSCI as a result of altered sensory inputs. Moreover, the excellent test–retest reliability of this characterization with the use of IMUs supports its effectiveness in assessing and monitoring balance-related changes in individuals with iSCI in both clinical research and practice settings.

To compare the consistency of findings using similar markers across different studies involving older adults, several conclusions have emerged. Many studies have found that COP-based time domain markers, especially COP mean velocity and path length, are effective in distinguishing age-related differences in static stability. Additionally, the diffusion function of the stabilogram has proven to be a reliable marker in multiple studies for identifying older adults. Studies that incorporate IMUs have also reached the conclusion that the RMS value of COM acceleration is a valuable marker for distinguishing the elderly from control groups. Furthermore, various studies employing both force plates and IMUs have reported a negative correlation between COM acceleration measures and COP-based measures with the clinical scale BBS. Similar to older adults, COP-based measures, notably COP mean velocity, have proven to be the most reliable and valid marker for distinguishing individuals with iSCI. Also, multiple studies involving individuals with iSCI have conducted the Limit of Stability test, one of them revealing that sway path length and time are highly correlated with the BBS scale. Furthermore, clinical scale assessments, such as the mini-BEST, have shown negative correlations with COP mean velocity, Romberg ratio of COP sway area, and RMS distance.

**Figure 4:**
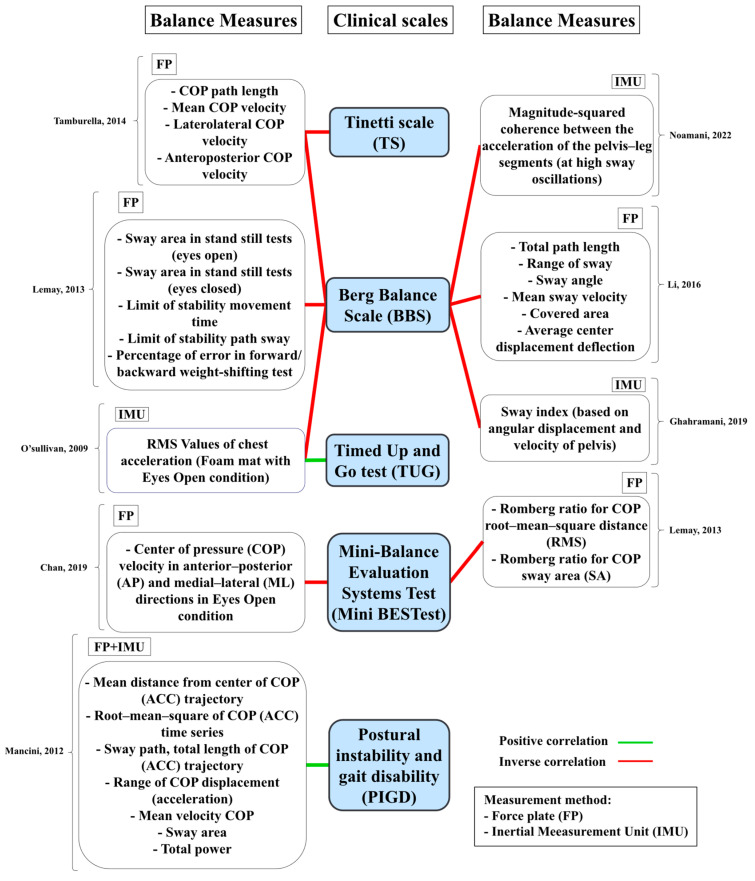
Correlations between clinical scales and balance measures investigated in the reviewed research papers [17,21,22,35,105,117,118,122,127,134].

Figure 4 provides a visual summary of the correlations between clinical scales and balance measures examined in the reviewed literature.

In summary, the review of balance assessment methods in older adults and individuals with iSCI highlights the value of utilizing both in-lab equipment and IMUs. While in-lab equipment provides precise measurements, the use of IMUs, although less explored, offers a portable and accessible option for regular testing in real-world clinical and rehabilitation settings. By combining the strengths of both approaches, comprehensive and objective evaluations of balance can be conducted, leading to improved understanding, more effective interventions, and enhanced outcomes for individuals with iSCI. Further exploration and utilization of wearable IMUs in balance assessment hold great potential especially for objective outcome evaluation of rehabilitative interventions. There is a need to investigate the feasibility of using IMUs integrated into the functional scale tests in a clinical setting for objective outcome evaluation. There are still several gaps and future areas of work in methodologies using wearable IMUs for objective balance assessment of older adults and individuals with iSCI. These include the need for standardization of protocols and metrics to ensure consistency across studies and enable reliable comparisons. Further validation studies are required to establish the accuracy and reliability of IMU-based methods, comparing them with gold-standard measures and assessing concurrent validity. Longitudinal studies are also necessary to track changes in balance over time and assess the effectiveness and sensitivity of wearable IMUs.

## 6. Existing Gaps and Future Directions

Despite the advancements in methodologies using wearable IMUs for objective balance assessment in older adults and individuals with iSCI, several gaps and future areas of research still exist. The standardization of protocols and metrics is necessary to ensure consistency across studies and facilitate reliable comparisons. Validation studies are essential to establish the accuracy and reliability of IMU-based methods by comparing them with gold-standard measures and assessing concurrent validity. Additionally, longitudinal studies are crucial to monitor changes in balance over time and assess the sensitivity and effectiveness of wearable IMUs.

Future research should focus on determining the sensitivity of instrumented tests with IMUs in detecting changes in balance biomarkers in response to specific rehabilitation programs among patients with impaired balance in clinical settings. Evaluating the effectiveness of targeted interventions to reduce future fall incidences and their adverse consequences among individuals with impaired balance is also crucial. Utilizing IMU-based objective measures to diagnose balance disorders that may be missed by traditional balance assessment tools, such as BBS, and determining underlying causes by comparing balance biomarkers of patients with those of able-bodied individuals can provide valuable insights for personalized therapy. Moreover, IMU-based objective balance assessment methodologies hold the potential for researchers to track subtle changes in a patient’s balance and monitor progress during rehabilitation. By obtaining balance biomarkers before and after interventions, researchers can objectively evaluate the effectiveness of the introduced interventions.

Furthermore, future studies should explore the use of IMUs for remote balance evaluation at patients’ homes, enabling remote health monitoring without requiring the physical presence of healthcare professionals. This is particularly advantageous in situations where professional resources are limited or during healthcare crises, such as the COVID-19 pandemic, when access to healthcare is a challenge for high-risk individuals with other underlying medical conditions. Emphasizing these areas of research will contribute to a more comprehensive understanding of traditional and IMU-based balance assessment in older adults and individuals with iSCI and enhance the potential for personalized interventions and remote health monitoring to improve overall balance and reduce fall risk in these vulnerable populations.

## 7. Conclusions

This paper presented a review of the literature on static balance, including objective assessment methodologies, using in-lab and wearable motion tracking systems, standing balance assessment for older adults, and post-iSCI in clinical research. This review revealed that many research studies utilized in-lab equipment, such as motion capture cameras and force platforms, in addition to conventional clinical scales, to obtain objective balance measures in older adults and ambulatory individuals with iSCI. However, the use of wearable IMUs as a convenient alternative with a higher level of clinical utility has been less investigated in these populations for the purpose of obtaining clinically meaningful measures of standing. Additionally, the use of wearable IMUs has not been extensively studied for the objective outcome evaluation of rehabilitative interventions in these populations. Future studies should determine the sensitivity of instrumented tests for detecting changes in balance biomarkers due to specific rehabilitation programs among patients with impaired balance in clinical settings. Also, future studies should evaluate the effectiveness of such targeted interventions to reduce future fall incidences and their adverse consequences among individuals with impaired balance. This review paper holds potential value for a diverse audience, especially researchers and healthcare professionals engaged in the study of static balance. This study offered a comprehensive analysis of static balance assessment, covering well-established posturography methods as well as the relatively less explored field of IMUs. Furthermore, it identified existing research gaps, providing guidance for future endeavors. This study can thus serve as an informative resource for readers seeking to select the most appropriate static balance assessment method for their specific application and environment. As a result, it can facilitate informed clinical decision-making for two distinct groups: older adults and individuals with SCI.

## Data Availability

No data were generated.
